# Performance Verification of High Sensitivity Analyzer for TAT, PIC, TM, and t-PAIC

**DOI:** 10.1055/a-2751-8459

**Published:** 2025-12-13

**Authors:** Yanhong Liu, Bo Guo, Guanghui Chen, Caixia Chen, Zhen Meng, Yan Xie, Yanru Fan, Rufei Ma, Lan Gao

**Affiliations:** 1 Department of Laboratory Medicine, Henan Provincial People's Hospital, People's Hospital of Zhengzhou University, People's Hospital of Henan University, Zhengzhou, Henan, China; 2 Department of Critical Care Medicine, Henan Key Laboratory for Critical Care Medicine, Zhengzhou Key Laboratory for Critical Care Medicine, Henan Provincial People's Hospital, People's Hospital of Zhengzhou University, People's Hospital of Henan University, Zhengzhou, Henan, China

**Keywords:** performance verification, thrombosis and hemostasis, thrombin–antithrombin complex, a2-plasmininhibitor–plasmin complex, thrombomodulin

## Abstract

**Background:**

Thrombin–antithrombin complex (TAT), a2-plasmininhibitor–plasmin complex (PIC), thrombomodulin (TM), tissue plasminogen activator–plasminogen activator inhibitor complex (t-PAIC) has been increasingly applied in clinical practice in recent years, especially in the diagnosis and treatment of diseases associated with thrombosis and hemorrhage. However, there is no universally accepted evaluation standard for the performance verification of these four indicators currently. Therefore, we designed experiments to verify the precision, trueness, carryover, linearity, and reference intervals of these four indicators. This study is expected to provide references for subsequent research in terms of data and experimental methods.

**Methods:**

According to the Clinical and Laboratory Standards Institute (CLSI) guidelines EP15-A2, EP06-A, and C28-A, the precision, trueness, carryover, linearity, and reference intervals were evaluated.

**Results:**

The within-laboratory CVs of TAT, PIC, TM, and t-PAIC were 3.67, 6.51, 3.64, and 2.46% on Control L and 4.68, 4.67, 5.08, and 3.87% on Control H. The assigned value of calibrations of TAT, PIC, TM, and t-PAIC were all included in the verification intervals. The biases of the four items of Calibration 1 were −6.67, −0.90, −3.58, and −6.78% and biases on Calibration 2 were −2.70, 1.63, 2.66, and −1.16%, respectively, compared with the assigned value provided by the manufacturer. The carryover rate of each indicator was less than 1%. Within the range that meets clinical use, the best fit curves of the four indicators were linear, and the correlation coefficients of all indicators were greater than 0.99. The reference intervals provided by the manufacturer were appropriate in our laboratory.

**Conclusion:**

The performance of HISCL-5000 analyzer for TAT, PIC, TM, and t-PAIC analysis were acceptable and the systems were suitable for clinical analysis.

## Introduction

Thrombotic diseases are a major health concern worldwide. This trend is likely to continue due to aging populations and increasing rates of obesity and sedentary lifestyles. 1 Laboratory testing plays a crucial role in diagnosis and management of thrombotic disorders. The early identification and accurate assessment of the initiation of the coagulation system and fibrinolysis system directly influence clinical decision-making and patient prognosis. Unfortunately, thrombin and plasmin have a short half-life in the body and are rapidly neutralized upon formation, making direct detection impossible. However, it was discovered that the complexes formed with antithrombin and antiplasmin, known as thrombin–antithrombin complex (TAT) and a2-plasmininhibitor–plasmin complex (PIC), can be detected to determine the levels of their production. Studies have shown that TAT is significantly increased in patients with deep vein thrombosis (VTE), acute myocardial infarction, and ischemic stroke, indicating its potential as a biomarker for the early diagnosis of these conditions. 2 Since D-dimer and fibrinogen degradation products (FDP) are products of fibrinolysis, elevations in PIC occur earlier than those of D-dimer and FDP. Studies have found that plasma levels of PIC are a useful biomarker for assessing the risk of VTE, and it can be used to determine whether orthopedic trauma patients require pharmacological prophylaxis. 3 Combined analysis of PIC and TAT can determine whether a patient is predominantly in hypercoagulable or hyperfibrinolytic state. 4

Thrombomodulin (TM), a protein secreted by endothelial cells, can form complexes with thrombin, which serves as a sensitive indicator of endothelial injury. Numerous studies have confirmed that TM levels can significantly increase in many inflammatory conditions. 5 Tissue plasminogen activator–plasminogen activator inhibitor complex (t-PAIC) is a complex formed when t-PA (tissue plasminogen activator) and PAI-1 (plasminogen activator inhibitor-1) are released into the blood during endothelial cell injury, which has high diagnostic value for severe infection. Studies have shown that t-PAIC levels in patients with septic shock are significantly higher than in patients with sepsis. 6 7 8 In addition, TAT, PIC, TM, and t-PAIC can predict the possibility of thrombosis at an early stage in the patients with COVID-19. 9

Performance verification of the analytical system is a prerequisite of accuracy and reliability of test results. There is limited research on evaluating the performance of these four indicators, and the performance requirements are not yet clear. In this study, based on the requirements of guidelines such as EP15-A2, 10 EP06-A, 11 and C28-A 12 issued by the Clinical and Laboratory Standards Institute (CLSI), and referencing the performance indicators provided by manufacturers, we conducted performance verification of precision, trueness, carryover, linearity, and reference intervals for TAT, PIC, TM, and t-PAIC.

## Materials and Method

### Samples

The samples used for precision verification were commercial control materials, the samples for trueness verification were traceable calibrators, and the samples for carryover, linearity, and reference intervals verification were plasma from patients and healthy people. The commercial control materials were processed according to the manufacturer's instructions, and clinical samples processed according to CLSI H21-A5. Venous blood was drawn into tubes containing 3.2% sodium citrate as the anticoagulant and was centrifuged at 1,500 g for 10 minutes. Samples with hemolysis, lipemia, and jaundice were removed. The study has been reviewed and approved by the Ethics Committee of Henan Provincial People's Hospital.

### Instrument and Reagents

Sysmex HISCL-5000 automatic coagulation analyzer and supporting reagents, including HISCL TAT Assay Kit (lot number SK0681), HISCL PIC Assay Kit (lot number SL0631), HISCL TM Assay Kit (lot number TG0661), HISCL t-PAIC Assay Kit (lot number TI0671), TAT Calibrator (SK0501), PIC Calibrator (K0501), TM Calibrator (SK0431), t-PAIC Calibrator (SK0471), TAT Control L (QTA-137), TAT Control H (QTA-237), PIC Control L (QPI-120), PIC Control H (QPI-220), TM Control L (QTM-122), TM Control H (QTM-222), t-PAIC Control L (QTP-119), t-PAIC Control H (QTP-219), and HISCL dilution (B0701), were all provided by Sysmex Company in Japan.

### Methods

#### Precision

Verification of repeatability and within-laboratory precision was performed over five consecutive days using two levels of commercial control materials in accordance with CLSI EP15-A2. Two levels of commercial control materials were prepared according to the manufacturer's instructions and analyzed three times. At least one level was at the medically determined level.

#### Trueness

Trueness was verified at the same time as precision verification. Two levels of traceable calibrators were analyzed in duplicate a day for a total of 5 days.

#### Carryover

For each item, two patient samples with high and low concentration were selected. Each sample was divided into three equal parts, and the samples were tested in the order of H1, H2, H3, L1, L2, L3. Then carryover rate (CR) was calculated. CR = (L1 − L3)/(H3 − L3) × 100%.

#### Linearity

The high concentration patient sample pool was prepared from 20 patients as Pool H and the low concentration sample was distilled water as Pool L. Six samples pool of equally spaced concentration were prepared according to the ratio of 5L, H ^+^ 4L, 2H ^+^ 3L, 3H ^+^ 2L, 4H ^+^ L, 5H. Each sample pool was analyzed three times.

#### Reference Intervals

A total of 20 samples from healthy people were used to verify the manufacturer's reference intervals according to C28-A. If no more than 2 of the 20 results fall outside the reference interval, the reference interval was considered acceptable. Once 3 or more results fall outside the range, another 20 samples were obtained and the analysis was repeated. If no more than two of these new results fall outside, the reference intervals were acceptable. However, if three or more again fall outside the range, laboratory established their own reference intervals.

### Statistical Analysis

#### Precision

Repeatability was represented by s _R_ and within-laboratory precision was represented by s _WL_ . The within-laboratory precision was expressed as a percentage of the mean ( *CV _WL_* ) for comparison with the precision required by the manufacturer in the laboratory ( *CV _WL-mfr_* ). The formulas were as follows:

where:
D = total number of days
n = total number of replicates per day
x _di_ = result for replicates per day (three replicates)
average of all results for day d
mean of all measurement results
If *CV _WL_* ≤ *CV _WL-mfr_* , then the manufacturer's claim for precision was verified.

#### Trueness

The verification interval (VI) and bias were calculated. If assigned value (AV) was included in the VI, then the trueness is verified.

where:
s _x_ = the standard deviation of all measurement results
s _x̄_ = standard error
x̄  = mean of all measurement data
s _a_ = combined standard uncertainty of reference material
N − 1 = degrees of freedom, α = assume a false rejection rate

If AV was included in the VI, then the trueness is verified.

#### Linearity

The mean value of the test value was taken as the measured concentration. The calculated value of each pool was calculated by the concentration of Pool H and Pool L, calculated value = (C _L_ × V _L_ + C _H_ × V _H_ )/(V _L_ + V _H_ ), in which the concentration of Pool H is C _H_ and the volume of Pool H used is V _H_ , likewise, the concentration of Pool L is C _L_ and the volume of Pool L used is V _L_ . Polynomial regression analysis was performed by SPSS and t-test was performed to determine whether the nonlinear coefficients of second and third polynomial models are significantly different from zero. Once the best-fitted curve was linear, the method was considered linear.

## Results

### Precision

The precision verification results are shown in Table 1 . The coefficient of variation of within-laboratory imprecision of TAT, PIC, TM, and t-PAIC were all below 10%, which meets the requirements of manufacturer.

**Table 1:** Precision verification of TAT, PIC, TM, and t-PAIC

	Control L					Control H				
		s _r_	s _WL_	* CV _WL_* (%)	* CV _WL-mfr_* (%)		s _r_	s _WL_	* CV _WL_* (%)	* CV _WL-mfr_* (%)
TAT	10.25	0.32	0.38	3.67	10	39.31	1.51	1.84	4.68	10
PIC	2.53	0.12	0.16	6.51	10	8.36	0.36	0.39	4.67	10
TM	22.07	0.36	0.80	3.64	10	91.89	1.81	4.67	5.08	10
t-PAIC	4.09	0.06	0.10	2.46	10	16.07	0.37	0.62	3.87	10

Abbreviations: *CV _WL_* , within-laboratory coefficient of variation; *CV _WL-mfr_* , the manufacturer's claimed within-laboratory coefficient of variation; PIC, a2-plasmininhibitor–plasmin complex; s _r_ , standard deviation of repeatability; s _WL_ , standard deviation of within-laboratory precision; TAT, thrombin–antithrombin complex; TM, thrombomodulin; t-PAIC, tissue plasminogen activator–plasminogen activator inhibitor complex;  , mean of all measurement results.

### Trueness

The verification range included the assigned value of calibrator; as shown in Table 2 , the trueness of TAT, PIC, TM, and t-PAIC were verified.

**Table 2:** Trueness verification of TAT, PIC, TM, and t-PAIC

		x̄	s _x_	VI	Assigned value	Bias (%)
TAT	Calibrator 1	2.94	0.24	0.99–4.89	3.15	−6.67
	Calibrator 2	6.13	0.09	4.19–8.07	6.3	−2.70
PIC	Calibrator 1	0.11	0.01	0.10–0.12	0.111	−0.90
	Calibrator 2	0.32	0.01	0.31–0.34	0.319	1.63
TM	Calibrator 1	40.98	1.47	39.34–42.62	42.5	−3.58
	Calibrator 2	101.43	2.92	98.37–104.49	104.2	−2.66
t-PAIC	Calibrator 1	2.82	0.24	−0.27–5.91	3.025	−6.78
	Calibrator 2	6.06	0.14	2.90–9.06	6.05	−1.16

Abbreviations: PIC, a2-plasmininhibitor–plasmin complex; s _x_ , standard deviation of test results; TAT, thrombin–antithrombin complex; TM, thrombomodulin; t-PAIC, tissue plasminogen activator–plasminogen activator inhibitor complex; VI, verification interval; x̄, mean of all measurement results.

### Carryover

The results of carryover verification are presented in Table 3 . The CR for all items was below 1%.

**Table 3:** Carryover verification of TAT, PIC, TM, and t-PAIC

	TAT	PIC	TM	t-PAIC
H1	116.80	38.500	206.700	105.600
H2	117.40	38.700	198.500	104.800
H3	115.90	40.870	197.600	107.400
L1	2.10	0.392	5.700	5.500
L2	2.10	0.376	5.700	5.500
L3	2.10	0.375	5.600	5.500
CR (%)	0	0	0.001	0

Abbreviations: CR, carryover rate; PIC, a2-plasmininhibitor–plasmin complex; TAT, thrombin–antithrombin complex; TM, thrombomodulin; t-PAIC, tissue plasminogen activator–plasminogen activator inhibitor complex.

### Linearity

**Fig. 1:**
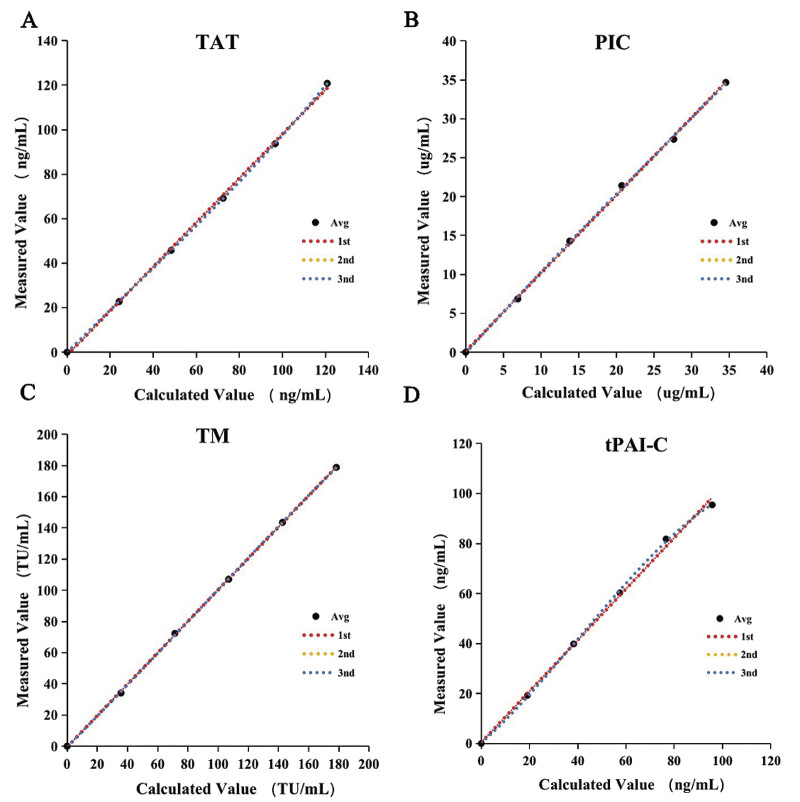
Polynomial regression analysis. ( **A** ) Polynomial regression of thrombin–antithrombin complex (TAT). ( **B** ) Polynomial regression of a2-plasmininhibitor–plasmin complex (PIC). ( **C** ) Polynomial regression of thrombomodulin (TM). ( **D** ) Polynomial regression of tissue plasminogen activator–plasminogen activator inhibitor complex (t-PAIC). r, correlation coefficient.

Polynomial regression results showed that the best fit curve of all four items was liner ( Fig. 1) . The linear range and correlation coefficients are shown in Table 4 .

**Table 4:** Linearity verification of TAT, PIC, TM, and t-PAIC

	Linear range	Linear equation	r
TAT	0–120	y = 0.9983x − 1.0068	0.9995
PIC	0–35	y = 0.9967x – 0.0987	0.9996
TM	0–175	y = 0.992x – 0.2571	0.9999
t-PAIC	0–95	y = 0.9875x + 0.4002	0.9985

Abbreviations: PIC, a2-plasmininhibitor–plasmin complex; r, correlation coefficient; TAT, thrombin–antithrombin complex; TM, thrombomodulin; t-PAIC, tissue plasminogen activator–plasminogen activator inhibitor complex.

### Reference Intervals

The reference intervals of TAT, PIC, and TM were verified using samples from 20 healthy individuals, including 10 male and 10 female. However, the reference interval of t-PAIC is different between male and female; samples from 20 male and 20 female were tested and calculated. The results of reference intervals verification showed that the number of samples beyond the reference range is less than 10% ( Table 5) .

**Table 5:** Reference intervals verification of TAT, PIC, TM, and t-PAIC

		Reference intervals	N _out-of-RI_	% _out-of-RI_
TAT		<4.0 ng/mL	1	5
PIC		<0.8 ug/mL	0	0
TM		3.8∼13.3 TU/mL	0	0
t-PAIC	Male	<17.0 ng/mL	0	0
	Female	<10.5 ng/mL	0	0

Abbreviations: N _out-of-RI_ , the number of samples outside the reference interval; PIC, a2-plasmininhibitor–plasmin complex; r, correlation coefficient; TAT, thrombin–antithrombin complex; TM, thrombomodulin; t-PAIC, tissue plasminogen activator–plasminogen activator inhibitor complex; %out-of-RI, percentage of samples outside the reference interval.

## Discussion

Repeatability is the disagreement among a set of replicate measurements when all measurements are made under the same conditions or within a single run of a procedure. Within-laboratory precision refers to the disagreement among replicate measurements over a long period of time, taking into account the main sources of errors within the laboratory. Within-laboratory precision reflects the accumulation of various error sources, including repeatability. 9 Our data are consistent with this, that is, s _WL_ is always greater than s _R_ . For TAT, PIC, TM, and tPAIC, the repeatability of high-level samples may not be as good as low-level samples. For these four novel indicators, there are currently no industry standards for performance verification. The verification criteria for within-laboratory precision in this study were provided by the manufacturer. We believe that a precision of less than 10% in the laboratory can meet the daily analysis requirements. We hope that our research can serve as a reference for future studies.

It is necessary to clarify the criteria for each validation indicator, which can be obtained by consulting the instructions or referring standards. In addition, evaluation criteria can be developed based on biological variation. The advantage of quality indicators determined by biological variations is that they are based on clinical research and consider the impact of measurement errors on the clinical interpretation of results, which helps in disease judgment and efficacy monitoring. 13 It should be noted that reliable biological variants should be used. 14 At present, there is no reliable biological variation data for TAT, PIC, TM, and tPAIC. EP15A2 considers both the repeatability of the measurement system and the uncertainty of the reference material in the trueness verification. Verification interval may be more suitable for TAT, PIC, TM, and tPAIC with multiple influencing factors and no reference method. If the precision verification is passed, but the trueness verification is not passed, compare bias with relevant industry standards. However, there is also a lack of bias evaluation criteria for TAT, PIC, TM, and tPAIC.

The linear range of TAT, PIC, TM, and tPAIC validated in this study can meet the needs of clinical screening for VTE patients and predicting prognosis. 15 16 We found that the reference intervals provided by the manufacturer are applicable to clinical practice. In healthy individuals, there is no activation of the coagulation and fibrinolysis systems, nor is there endothelial damage. Therefore, TAT, PIC, TM, and t-PAIC are generally at a lower level. However, the normal value of t-PAIC varies between men and women, and different reference intervals should be set according to sex.

The performance of the measurement system can meet clinical needs, and reliable biological variation data and evaluation criteria for TAT, PIC, TM, and t-PAIC need to be studied.

## Notes

**What is Known About This Topic?**

- The analyses of TAT, PIC, TM and t-PAIC plays a crucial role in diagnosis and management of thrombotic disorders.
- Only a few studies have investigated the performance verification of TAT, PIC, TM and t-PAIC according to the calculation methods in CLSI related documents.

**What Does This Paper Add?**

- This study evaluates the performance of TAT, PIC, TM and t-PAIC according to CLSI related documents in a standardized manner.
- This study is the first to evaluate the truness of TAT, PIC, TM and t-PAIC.
